# Transcriptional and cytokine signatures of *Mycobacterium abscessus* complex pulmonary disease during disease progression and treatment

**DOI:** 10.1371/journal.pntd.0012943

**Published:** 2025-03-31

**Authors:** Ayesa Syenina, Yi Hern Tan, Danny Jian Hang Tng, Sandy Xue Qi Sim, Valerie Shyn Yun Chew, Jia Xin Yee, Eugenia Ziying Ong, Eng Eong Ooi, Jenny Guek Hong Low, Dorothy Hui Lin Ng

**Affiliations:** 1 Viral Research and Experimental Medicine Centre (ViREMiCS), SingHealth Duke-NUS Academic Medical Centre, Singapore, Singapore; 2 Programme in Emerging Infectious Diseases, Duke-NUS Medical School, Singapore, Singapore; 3 Department of Respiratory Medicine and Critical Care, Singapore General Hospital, Singapore, Singapore; 4 Department of Infectious Diseases, Singapore General Hospital, Singapore, Singapore; 5 Department of Clinical Translational Research, Singapore General Hospital, Singapore, Singapore; Aarupadai Veedu Medical College & Hospital, INDIA

## Abstract

**Background:**

*Mycobacterium abscessus* complex pulmonary disease (MABC-PD) is a chronic and often relapsing disease with considerable morbidity, especially among individuals with other chronic pulmonary conditions. A major clinical challenge lies in distinguishing infection-related symptoms from underlying lung disease and identifying reliable prognosticators to guide treatment decisions and monitoring therapeutic response.

**Methodology/Principal Findings:**

To address the gaps in clinically relevant indicators, we profiled whole blood transcriptome and 45 plasma proteins of MABC-PD patients across different disease and treatment phases. Whole blood bulk RNA-sequencing revealed that MABC-PD patients with progressive disease exhibited elevated expression of genes related to innate immune and inflammatory responses, with reduced abundance of genes associated with peripheral T and NK cells. Among the 45 plasma cytokines and chemokines profiled, plasma levels of TNFSF10 were significantly reduced, while IFNγ, interleukin-17F (IL17F) and IL17C were elevated in patients with disease progression, despite the reduced abundance of peripheral T and NK cell-associated genes, suggesting recruitment of activated T cells to infection sites in the lungs during disease progression. Receiver operating characteristic (ROC) curve analysis of IFNγ and IL17F demonstrated strong predictive performance for differentiating patients with disease progression from healthy controls, with AUCs of 0.946 (95% CI 0.829-1.000) and 0.875 (95% CI 0.6699-1), respectively.

**Conclusions:**

These findings provide insights into the immune profiles of MABC-PD patients during disease progression and suggest that T cell-associated cytokines, such as IFNγ and IL17F, could serve as useful biomarkers for identifying those under watchful waiting or post-treatment who are at risk of disease progression, thereby aiding in more timely and targeted therapeutic interventions.

## Author summary

*Mycobacterium abscessus* complex pulmonary disease (MABC-PD) is a chronic, relapsing lung infection that causes significant health problems, especially in patients with preexisting lung conditions. Distinguishing infection-related symptoms from those caused by underlying lung disease presents a major clinical challenge as there are currently no useful biomarkers available to guide treatment decisions and assess response to treatment. In this study, we analyzed the blood immune profiles of MABC-PD patients across different stages of the disease and treatment. Our findings showed that patients with disease progression during intensive phase treatment had increased activity in genes related to the innate immune response and inflammation, but reduced activity in genes associated with T and NK cells. Out of the 45 immune proteins we measured in the blood, only four were significantly altered in patients with disease progression: TNFSF10 was reduced, and IFNγ, IL17F and IL17C were elevated. This pattern suggests that T cells were being activated and likely recruited to infection sites in the lungs. Using ROC curve analysis, we found that the levels of IFNγ and IL17F could predict for relapse. These findings provide insights into the immune response in MABC-PD and suggest that IFNγ and IL17F could be useful biomarkers for identifying patients at risk of disease progression, aiding in earlier and more targeted interventions.

## Introduction

Non-tuberculous mycobacteria pulmonary disease (NTM-PD) is a growing global health concern [1,2], characterized by recurrent or relapsing pulmonary disease that contribute to cumulative morbidity, structural lung damage, and a substantial healthcare burden [3–6]. Over the past two decades, its incidence and prevalence have been rising steadily, even surpassing tuberculosis in some developed countries [7–9]. The *Mycobacterium abscessus* complex (MABC), comprising three subspecies: *abscessus*, *bolletii*, and *massiliense* [10], is one of the most clinically significant NTM species due to its association with treatment challenges [11,12].

Managing MABC pulmonary disease (MABC-PD) is notoriously difficult due to its high rates of antibiotic resistance and poor correlation between antibiotic susceptibility testing and treatment outcomes. Current guidelines recommend a phased approach, beginning with an intensive phase involving at least three drugs (intravenous, inhaled and/or oral) followed by a prolonged continuation phase of oral and/or inhaled antibiotics, typically lasting over a year [13]. However, these regimens are poorly tolerated, and treatment durations often vary significantly in clinical practice [14–16]. Furthermore, treatment success is usually assessed through culture conversion and improvements in symptoms or quality-of-life, all of which are metrics that are difficult to implement in practice; culture conversion requires multiple sputum samples, which can be challenging to obtain from patients who are unable to expectorate sputum. Alternative methods, such as bronchoalveolar lavage, are invasive and not routinely used in clinical care [13,17]. Additionally, nearly half of MABC-PD patients fail to achieve sputum conversion or experience meaningful symptom or quality-of-life improvements [4,18,19], leaving their optimum treatment strategies uncertain. For these patients, multiple treatment courses may be administered based on symptoms or radiological changes, even though differentiating MABC-PD symptoms from those of coexisting lung conditions is difficult [20]. While chest computer tomography (CT) scans can aid in disease monitoring, frequent imaging involve radiation risks, and radiological changes do not always correlate with clinical outcomes [21].

Complicating case management even further is that not all newly diagnosed MABC-PD patients require treatment despite sputum positivity; some are managed through watchful waiting [5]. However, identifying appropriate candidates for this approach remains challenging, as clinicians must balance the risks of disease progression with potential antibiotic side effects. The lack of specificity of symptoms, sputum results and imaging findings, also complicates clinical decision-making for treatment initiation.

The subjectivity inherent in treatment initiation and monitoring outcomes highlight the need for novel management strategies. Advancements in immune profiling has deepened our understanding of the molecular interplay during active and latent tuberculosis [22, 23], and could offer insights into MABC-PD [24]. For example, Marty *et al* demonstrated that patients with progressive NTM-PD, including a small number with MABC-PD, exhibited elevated peripheral blood T cell responses compared to non-progressive disease using flow cytometry and ELISpot [25]. However, this study included a mix of NTM species, including *Mycobacterium avium* (n=13), *M. abscessus* complex (n=3), *M. chelonae* (n=1) and *M. kansasii* (n=1), potentially introducing heterogeneity in disease progression and severity due to species-specific immune responses. Focusing exclusively on MABC-PD – the most clinically significant NTM in our region [26–28] – could provide greater resolution in understanding its underlying immune response and help identify biomarkers to guide patient management.

In this cross-sectional study, we analyzed MABC-PD patients using whole blood transcriptomic and cytokine/chemokine profiling to identify immune markers associated with different phases of MABC-PD: intensive, continuation, post-treatment and watchful waiting phases. As a proof-of-concept, we applied these immune markers to a group of patients on watchful waiting and who were post-treatment to assess the potential of these biomarkers for identifying patients who are at risk of disease progression or relapse.

## Methods

### Ethical Approval statement

This study was approved by the SingHealth Centralized Institutional Review Board (ID: 2022-2151) and the National University of Singapore Institutional Review Board (ID: 2022-318). Written formal consent was obtained from study participants.

### Study design

Patients from the Singapore General Hospital were assessed for eligibility and recruited upon informed consent, if they met the following inclusion criteria: a previous diagnosis of MABC-PD as defined by the 2007 ATS/IDSA guidelines [29], and age ≥21 years. Exclusion criteria included conditions such as human immunodeficiency virus (HIV) infection, malignancy, bone marrow or solid-organ transplantation, primary or secondary immunodeficiency, use of steroids or biologics, cystic fibrosis, chronic obstructive pulmonary disease, emphysema, anti-1-alphatrypsin deficiency, idiopathic pulmonary fibrosis, active smoking, or pregnancy. Healthy controls were either healthcare workers or household contacts of patients, with no chronic respiratory symptoms or known history of NTM-PD. Whole blood and plasma samples were collected for RNA-sequencing analysis and cytokine analysis respectively.

### RNA-sequencing for gene expression analysis

Whole blood samples were collected in Tempus blood RNA tubes (Thermo Fisher Scientific, USA). RNA isolation was performed using the Tempus Spin RNA Isolation Kit (Thermo Fisher Scientific) according to the manufacturer’s protocol. RNA was sent to Azenta Life Sciences for sequencing using the Illumina Novaseq platform. Data processing was conducted using DESeq2 [30]. Data was pre-filtered to remove genes with low counts, specifically those with counts of less than 10 in more than 10 samples. Variance stabilizing transformation (VST) was applied on filtered data for principal component analysis (PCA) to assess variability and clustering among samples. Differential expression analysis between different phases of MABC-PD and healthy controls was performed using DESeq2 [30]. Genes with an adjusted *p-value* <0.05 and a fold-change of >1.3 and <‒1.3 were considered significantly upregulated or downregulated, respectively. An MA-plot was generated using DESeq2 [30] to visualize the mean of normalized counts across all samples for each gene and the log-fold-change between the different phase of MABC-PD and healthy controls. Gene-set enrichment analysis (GSEA) on of differentially expressed genes (DEGs) was conducted using the R package EnrichR v.3.2 [31] with BTM-plus [32].

Weighted gene co-expression network analysis (WGCNA) [33] was conducted on VST-transformed data. Briefly, WGCNA performs Pearson’s correlation between genes across the different samples measure and assigns correlation score of each gene pair. A soft threshold of 12 was selected based on the diagnostic graphs (S1A-B) for gene correlation network construction. Hierarchical clustering was performed on gene correlation network data to determine different gene modules. Subsequently, module-trait relationships, with MABC-PD disease stages as the trait being assessed, was conducted. Pathway enrichment analysis was conducted using the R package EnrichR v.3.2 [31] with BTM-plus [32]. Network plots of enriched genesets were generated using the cnetplot function from the R package EnrichR. To estimate immune cell populations, immune deconvolution was conducted on DESeq2 normalised RNA-sequencing data using the quantiTIseq [34] and Microenvironment Cell Populations (MCP)-counter [35] methods using the R package immunedeconv v.2.1.0. Box and whiskers plots were generated using R package ggplot2 v.3.5.1 and ggpubr v.0.6.0 for statistical comparison between groups. All plots and graphs were generated on RStudio v.2024.04.2 using R v.4.3.1.

### Proteomics analysis

Whole blood samples were collected in EDTA blood tubes (BD Vacutainer). To collect plasma, blood tubes were centrifuged at 2500rpm for 10 minutes at room temperature. The plasma (top aqueous layer) was then collected. Plasma samples were then used for proteomics analysis with Olink Target 48 Cytokine panel. Briefly, 45 oligonucleotide labeled antibody probe pairs were hybridized with plasma samples at 4°C overnight to form 45 unique DNA reporter sequences. These DNA reporter sequences were then amplified by PCR before being detected and quantified with the Olink Signature Q100, with readouts in pg/mL. Data analysis to box and whiskers plots were generated using R package ggplot2 v.3.5.1 and ggpubr v.0.6.0 for statistical comparison between groups, on RStudio v.2024.04.2 using R v.4.3.1. Multiple logistic regression was performed on GraphPad Prism v.10.4.1.

### Statistical analysis

Statistical analysis was performed on RStudio v.2024.04.2 and GraphPad Prism v.10.4.1. For categorical variables, statistical analysis was done with the Chi-square (χ2) test. For continuous variables, normality was assessed using skewness and kurtosis statistics. Parametric continuous data was assessed using the Student’s *t*-test. Nonparametric continuous data was assessed using the Kruskal-Wallis test. For all datasets, a *p*-value of less than 0.05 was considered significant.

## Results

### Characteristics of study subjects and study design

**Fig 1:**
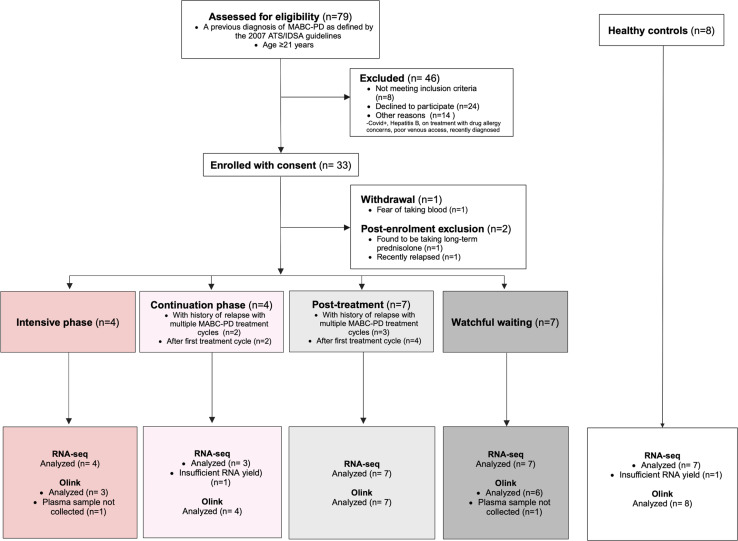
Consort diagram of patient recruitment.

An overview of the patient enrollment process is shown in Fig 1. A total of 33 patients were recruited between April 2022 and April 2023, with one withdrawal and two exclusions post-enrollment (Fig 1). MABC-PD patients were categorized based on treatment phase at recruitment: those with disease progression undergoing intensive phase treatment (n=4), continuation phase treatment (n=4), or in remission post-treatment (n=7). A separate group of MABC-PD patients on watchful waiting (WW) (n=7), who were managed conservatively without antibiotics, were also recruited. Healthy controls (n=8) were either healthcare workers or household contacts of patients with no chronic respiratory symptoms or known history of NTM-PD.

Patient and control demographics are shown in S1 Table; there were more female subjects in the patient groups compared to healthy controls. Patients in intensive phase/continuation phase treatment had a lower body mass index (BMI) compared to patients who were post treatment, on watchful waiting or healthy controls. There were no significant differences among patient and control groups in terms of age, ethnicity, tuberculosis history, diabetes mellitus, or radiological features. Patient treatment details are shown in S2 Table. Of the 4 patients on intensive phase treatment, all had disease progression after prior MABC-PD treatment cycles. Of the 4 patients on continuation phase treatment, 2 had disease progression after prior MABC-PD treatment cycles, and 2 were newly diagnosed MABC-PD patients on their first treatment cycle. Of the 7 patients in remission post-treatment, 3 had a history of disease progression with multiple MABC-PD treatment cycles, and 4 had completed their first treatment cycle, with enrollment occurring between 443 to 2410 days post-treatment.

A single peripheral blood draw from patients and controls was carried out at enrollment for cross-sectional bulk RNA sequencing and Olink immune proteomic analysis.

### Increased innate immune and inflammation gene expression and lower T and NK cell gene expression in patients with progressive disease

**Fig 2:**
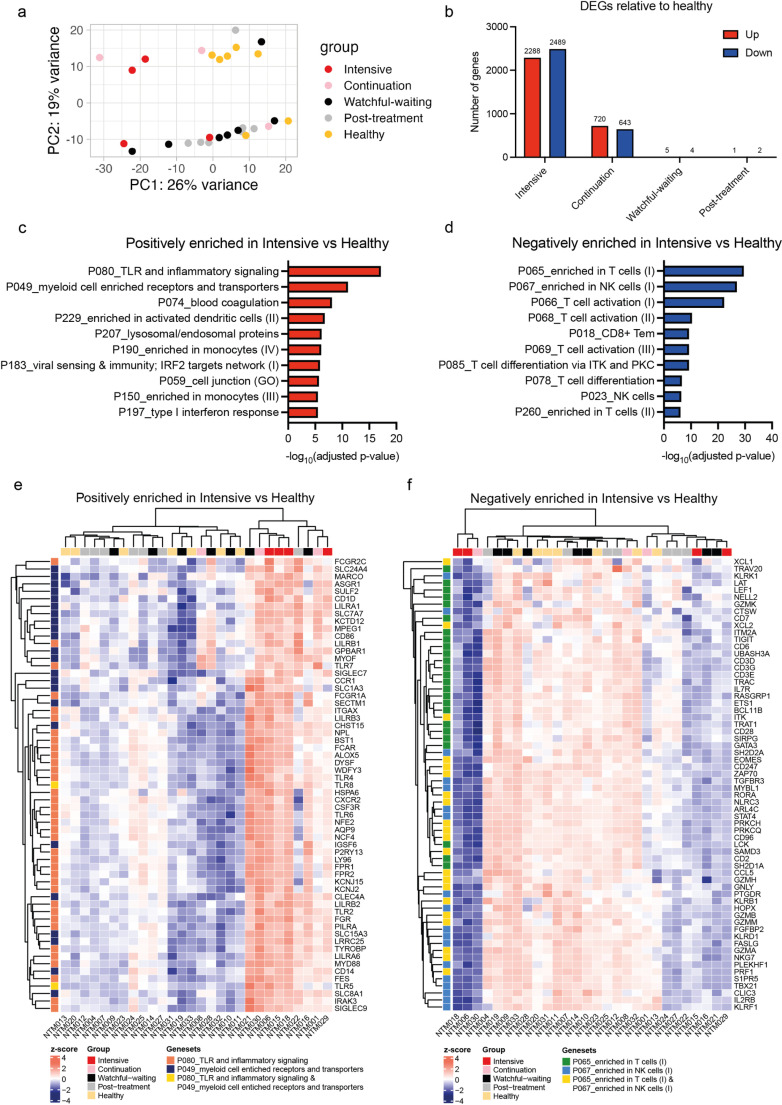
Increased expression of genes associated with the innate immune response and inflammation, and reduced expression of genes associated with T and NK cell functions in patients on intensive phase treatment. (a) Principal component analysis (PCA) of gene expression data derived from RNA-sequencing. Principal component (PC1) and PC2 accounts for 26% and 19%, respectively, variance of dataset. Each dot represents one patient from the study; different colours represent different phases of MABC-PD treatment and disease progression. (b) Differential expression analysis was conducted using Deseq2, imposing a threshold of adjusted *p-value* <0.05 and fold change of >1.3 and <-1.3. The number of differentially expressed genes (DEGs) in different phases of MABC-PD treatment and disease progression relative to healthy controls is presented. (c-d) Enrichr geneset pathway enrichment analysis (GSEA) using the BTM-plus module was conducted for identified DEGs. Pathways (c) positively and (d) negatively enriched in MABC-PD patients on intensive phase treatment compared to healthy controls. (e-f) Unsupervised clustering of leading-edge genes (LEGs) from the top two (e) positively and (f) negatively enriched genesets in MABC-PD patients on intensive phase treatment compared to healthy controls. Z-score of VST normalized gene counts are presented.

To understand host responses associated with MABC-PD disease progression, we analyzed the transcriptomic differences between patient groups and healthy controls. Transcriptomic data were available for all 4 intensive phase patients, 3 of 4 continuation phase patients (one excluded due to insufficient RNA yield), all 7 post-treatment patients, all 7 watchful waiting patients, and 7 of 8 healthy controls (one excluded due to insufficient RNA yield) (Fig 1). PCA revealed distinct gene expression patterns, particularly in intensive phase patients (PC1) and healthy controls (PC2), compared to other patient groups (Figs 2A and S2A-B). Concordantly, differential gene expression analysis between patient groups and healthy controls using DESeq2 [30] showed that intensive phase patients exhibited the most extensive transcriptomic changes (2288 upregulated and 2489 downregulated genes) (Figs 2B and S2C), followed by continuation-phase patients (720 upregulated and 643 downregulated genes) (Figs 2B and S2D). Watchful waiting and post-treatment groups showed minimal differential expression, with gene profiles resembling healthy controls (Figs 2B and S2E-F).

Geneset enrichment analysis on differentially expressed genes (DEGs) indicated that upregulated genes in both intensive and continuation-phase patients were associated with inflammatory and innate immune responses, including dendritic cell and monocyte-related genes. Conversely, downregulated genes were associated with T and NK cells (Fig 2C-D). A closer look at DEGs from the top two positively and negatively enriched genesets in intensive phase patients showed that patients on intensive phase treatment clustered entirely separate from healthy controls (Figs 2E-F and S2D). While most of the patients on watchful-waiting clustered with healthy controls, two out of seven clustered with the intensive phase group (Fig 2E-F). One post-treatment patient clustered with the intensive phase group, while the remainder clustered with healthy controls (Fig 2E-F).

To corroborate our differential gene expression analysis, we employed a secondary computational method, WGCNA [33,36], which reduces data dimensionality by clustering highly correlated genes into module eigengenes (ME) (S3A Fig). MEs that correlated significantly to various disease or treatment phases were subjected to gene set enrichment analysis (GSEA) using Enrichr [31] with established blood transcriptomic modules (BTM-plus) [32]. We identified six highly correlated MEs, that were associated with patients on intensive phase treatment, two MEs with patients on continuation phase, and one ME with post-treatment patients (S3B Fig). Three MEs were positively correlated to patients on intensive phase treatment: ME turquoise which included genes related to neutrophils, platelet activation, and toll-like receptor (TLR) and inflammatory signaling (S3C Fig); ME brown which included genes associated with macrophages and monocytes (S3D Fig); and ME black which comprised genes associated with antigen presentation, viral sensing and immunity, as well as type-1 interferon response (S3E Fig). Conversely, three other MEs were positively correlated: ME royal blue which included genes related to NK cells, T cell signaling, glycerophospholipid metabolism, and the extracellular matrix (S3F Fig); ME blue which included T and NK cell-associated genes (S3G Fig); and ME red which included genes associated with intracellular transport and ubiquitination (S3H Fig).

Additional gene modules were associated with MABC-PD patients in the continuation phase and post-treatment: ME dark red, ME yellow, and ME dark green. ME dark red included genes related to dendritic cells, eosinophils, and mast cells (S3I Fig). ME yellow included genes associated with the regulation of transcription and the CORO1A-DEF6 network (S3J Fig). ME dark green included genes associated with mast cells (S3K Fig).

Collectively, the transcriptomic data suggest significant perturbations in both innate and adaptive immune responses in patients on intensive phase treatment.

### Reduced abundance of NK and T cells in the peripheral blood in patients with progressive disease

To determine whether the observed reduction in NK and T cell gene transcripts were due to decreased peripheral blood cell counts or reduced expression, we employed previously established methods, quanTIseq [34] and Microenvironment Cell Populations-counter (MCP-counter) [35], to quantify immune cell types from our transcriptomic data. Both methods consistently revealed significant reductions in NK cells, T regulatory cells, CD4+ and CD8+ T cells abundance between patients in different treatment groups, with the most pronounced decreases in those undergoing intensive phase treatment (S4A-B Fig). These findings suggest that the reduced expression of NK- and T-cell associated genes could be attributed to the lower peripheral blood abundance of NK and T cells during disease progression.

### Elevated peripheral blood T cell-associated cytokine levels in progressive disease

**Fig 3:**
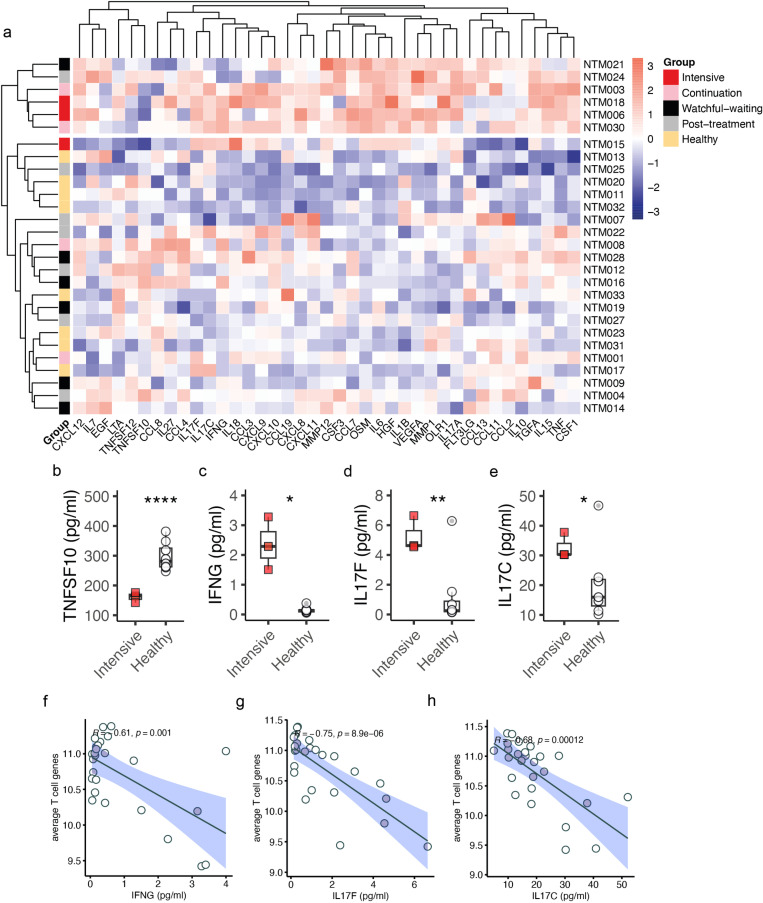
Elevated plasma concentrations of TNFSF10, IFN **γ,** **IL17F, and IL17C in MABC-PD patients on intensive phase treatment compared to healthy controls.** (a) Unsupervised clustering of plasma concentration of proteins in different treatment phases of MABC-PD. Z-scores of plasma concentrations are presented. (b-e) Box and whiskers plot comparing plasma concentration of (b) TNFSF10, (c) IFNγ, (d) IL17F, and (e) IL17C between patients on intensive phase treatment and healthy controls. Student’s *t*-test was used to test the difference in mean between the two groups. (e-f) Pearson’s correlation between (f) IFNγ, (g) IL17F, and (h) IL17C plasma concentration and average VST-transformed counts of T cell associated genes. ***n*** *= 3* (intensive phase); ***n*** = 8 (healthy controls).

**Fig 4:**
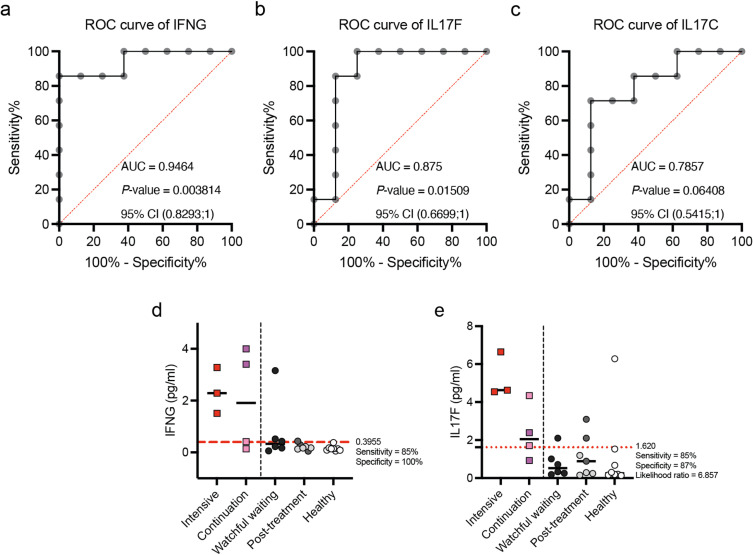
Immune profiling discriminates MABC-PD patients on treatment from healthy controls. Receiver operating characteristic (ROC) analysis was conducted with treatment group classified as MABC-PD patients on intensive and continuation phase treatment; no treatment group included healthy controls. (a-c) ROC curve of (a) IFNγ, (b) IL17F, and (c) IL17C. (e-f) Dotplot of plasma concentration of (e) IFNγ and (f) IL17F in patients at different phases of MABC-PD treatment, patients on watchful waiting, and healthy controls. The red horizontal line depicts cutoff values as defined by ROC analysis. Darker shades of pink and grey points depict relapsed MABC-PD patients who have previously received multiple treatment courses; lighter shades of pink and grey points depict MABC-PD patients in their first treatment course.

Thus far, our gene expression data indicated heightened innate immune activation and reduced peripheral NK and T cells. To build upon these findings, we measured the absolute concentration of 45 plasma chemokines and cytokines using the multiplex Olink Target 48 Cytokine panel across various treatment phases. Unsupervised clustering of the cytokine data clearly segregated all but one intensive phase patient from healthy controls (Fig 4A). Similarly, all but one patient in the watchful-waiting and post-treatment groups clustered with healthy controls (Fig 3A). Half of the patients (2/4) on continuation phase treatment clustered with healthy controls, and the other half with patients on intensive phase treatment (Fig 3A).

Among the 45 plasma chemokines and cytokines, TNFSF10, also known as tumour necrosis factor-related apoptosis-inducing ligand (TRAIL), was significantly decreased in patients on intensive phase treatment (Fig 3B). Conversely, IFNγ, interleukin-17F (IL17F), and interleukin-17C (IL17C), cytokines produced by activated T cells, were significantly increased in the same patient group (Fig 3C-E). Pearson’s correlation analysis further revealed that the concentration of IFNγ, IL17F, and IL17C negatively correlated with transcript levels of T cell associated genes (Fig 3F-H). These findings collectively suggest that in patients with disease progression, activated T cells may migrate from the peripheral blood to infection sites, leading to reduced peripheral T cell abundance.

### IFNγ and IL17F are potential biomarkers for MABC-PD treatment initiation

Given that cytokine measurements may potentially be more accessible to routine diagnostic labs compared to gene expression analysis, we next evaluated the potential of IFNγ, IL17F, and IL17C as biomarkers to identify patients with disease progression. These cytokines are associated with T cell activation and may indicate immune responses relevant to disease status. We conducted receiver operating curve (ROC) analyses to assess the sensitivity and specificity of these cytokines in distinguishing patients undergoing treatment (either intensive or continuation phase) from healthy controls (Fig 4A-C). We found that both IFNγ and IL17F significantly distinguished these groups, with area under the curve (AUC) values of 0.9464 (95% CI 0.8293-1) and 0.875 (95% CI 0.6699-1), respectively (Fig 4A-B). In contrast, IL17C had an AUC of 0.7857 (95% CI 0.5415-1), which was not statistically significant (Fig 4C).

To establish optimal cut-off values, we applied thresholds from our ROC analysis. For IFNγ, a cut-off of 0.3955pg/ml resulted in sensitivity of 85% and specificity of 100% (Fig 4D). For IL17F, a cut-off of 1.620pg/ml gave a sensitivity of 85% and specificity of 87%, with a likelihood ratio (LR) of 6.857 (Fig 4E). Applying these thresholds, we observed that these cytokine levels clearly distinguished patients on intensive phase treatment from healthy controls. Notably, among patients under watchful waiting and post-treatment, some resembled healthy controls, while others exceeded these cut-off values, resembling patients undergoing intensive phase treatment (Fig 4D-E).

Collectively, our findings suggest that IFNγ and IL-17F could potentially serve as biomarkers for identifying MABC-PD patients who may benefit from treatment, and differentiate them from those who can be managed with continued watchful waiting.

## Discussion

Non-tuberculous mycobacterial pulmonary disease (NTM-PD) is a chronic lung disease with rising prevalence, posing significant management challenges. Clinical decision-making is often hindered by nonspecific and insidious indicators such as radiological or symptomatic changes. Identifying patients most likely to benefit from treatment and determining the optimum duration of treatment remain critical, yet highly subjective, challenges.

In this study, we identified distinct gene expression and cytokine profiles associated with different treatment phases of MABC-PD. Patients undergoing intensive phase treatment exhibited increased expression of genes associated with neutrophil, macrophage and monocyte activation, as well as a hyperinflammatory response involving TLR and inflammatory signaling and a type I interferon response. Conversely, genes associated with T and NK cells were downregulated, reflecting a reduced peripheral abundance of these immune cells in blood. This immune profile mirrors findings in Mycobacterium *avium complex* (MAC)-PD [37, 38], suggesting shared immunopathogenic mechanisms between these NTM species.

Despite the reduced T cell-associated gene expression and their diminished peripheral abundance, elevated levels of IFNγ, IL17F and IL17C – cytokines produced by activated T cells – argue against T cell exhaustion as a primary explanation [39]. Instead, these findings suggest robust T cell activation and potential relocalization to the infection sites in the lungs [40]. IFNγ is produced primarily by T helper 1 (Th1) cells, cytotoxic T cells, and NK cells, and is crucial in the immune response against mycobacterial infections by activating macrophages, enhancing their ability to phagocytose and kill mycobacteria, and promotes the formation of granulomas to contain the infection [39]. Elevated IFN-γ levels in our intensive phase patients may indicate an active immune response aimed at controlling MABC-PD. IL-17 is produced predominantly by T helper 17 (Th17) cells and is integral to the host defense against mycobacterial infections by mediating neutrophil recruitment and promoting pro-inflammatory responses [41–43]. However, their overproduction may exacerbate inflammation, contributing to tissue damage and disease progression, as observed in other chronic infections and inflammatory lung diseases such as chronic obstructive pulmonary disease (COPD) and severe asthma [44]. Increased monocytic gene expression in patients on intensive phase treatment aligns with monocytes’ role in antigen presentation and facilitating T cell activation. However, the functional role of monocytes and T cells in MABC-PD remains uncertain; whether they primarily contribute to disease progression through exacerbating inflammation or protection by enhancing mycobacterial clearance cannot be gleaned from this study. Direct lung-specific analysis, such as bronchoalveolar lavage (BAL) fluid studies, could provide insight into immune activities and localization at the infection site. For example, BAL studies in tuberculosis have revealed distinct T cell phenotypes in the lungs compared to peripheral blood, with localized effector functions that could be difficult to be inferred from blood analyses alone [45,46]. While such studies are clinically challenging to conduct, they may be essential to unravel the precise immune interplay in MABC-PD pathogenesis.

Additionally, we found significantly reduced TNFSF10 levels in patients on intensive phase treatment. TNFSF10, also known as TRAIL, is a pleiotropic cytokine that regulates apoptosis, immune responses and inflammation. In lung diseases, TNFSF10 has been implicated in both protective and pathogenic processes, depending on the context and cellular targets [47]. For example, TNFSF10 can promote apoptosis of infected macrophages, aiding bacterial clearance [48], but may also contribute to tissue damage by inducing excessive cell death in epithelial and immune cells. In the context of MABC-PD, reduced TNFSF10 levels may reflect impaired apoptotic clearance of infected cells or dysregulated immune signaling [49]. Further studies are required to delineate the mechanisms by which TNFSF10 influences disease progression and its potential utility as a prognostic or therapeutic target in MABC-PD.

Despite these uncertainties, our findings suggest that T-cell associated immune responses, particularly reflected in IFNγ and IL17F levels, could serve as biomarkers to guide treatment decisions. Interestingly, patients on watchful waiting and post-treatment exhibited heterogenous immune profiles. Some patients showed evidence of T cell activation and inflammation despite variable clinical and radiological signs of disease progression. This discrepancy highlights the potential of immune profiling to provide a more nuanced basis for initiating or withholding treatment in such patients. Longitudinal follow-up studies are needed to determine whether these distinct immune profile correlate with disease progression or stability.

There are several limitations to our study. Firstly, it is a cross-sectional study, and our results only captured a single timepoint of the disease. Secondly, the relatively small sample size may also limit the sensitivity of our findings, and the analysis was limited to peripheral blood, with no direct comparison with lung samples. Thirdly, at our centre, MABC subspecies identification is only performed upon clinician request. As a result, we were not able to assess possible differences in immune profiles between subspecies. Finally, although this study was conducted in a single tertiary care center, variations in physicians’ recommendations and patients’ preferences could have influenced treatment decisions and the timing of antimicrobial initiation. Therefore, further validation in larger and more diverse cohorts with longitudinal follow-up is necessary to track immune signature dynamics over time and correlating these with disease progression or treatment.

In conclusion, findings from this study provide insights into the immune response in MABC-PD patients and suggest the use of IFNγ and IL17F levels as biomarkers to personalize MABC-PD treatment strategies. Further longitudinal studies are necessary to validate these findings and to explore their utility in clinical practice.

## Supporting information

S1 Table Characteristics of patients and controls. (XLSX)

S2 Table Treatment details of individual patients. (XLSX)

S1 Fig Determination of soft threshold for WGCNA. Diagnostic plots depicting (a) scale independence and (b) mean connectivity in determining soft threshold to be applied to WGCNA.
(TIF)

S2 Fig Differential expression analysis of RNA-sequencing data. (a-b) Heatmap of the top 50 genes in (a) PC1 and (b) PC2. Each column represents a single patient in the study, rows represent genes. Different phases of MABC-PD treatment and disease progression are depicted in different colours. Z-scores of normalised counts are presented. (c-f) MA-plot depicting the mean of normalized counts across all samples for each gene and the log-fold-change between MABC-PD patients (c) on intensive phase treatment, (d) continuation phase, (e) on watchful waiting, and (f) post-treatment relative to healthy controls.
(TIF)

S3 Fig WGCNA. (a) Gene dendogram obtained by hierarchical clustering of dissimilarity based on Topological Overlap. (b) Heatmap of weighted gene co-expression network analysis (WGCNA) module-trait relationship, with trait being different treatment phases of MABC-PD. Rows represent module eigengenes that summarize gene modules defined in the hierarchical clustering analysis. Columns represent the different traits (i.e., treatment phases of MABC-PD). Values indicated in the box are the correlation coefficients with asterisks indicating the p-values. (c-k) Enrichr geneset pathway enrichment analysis using the BTM-plus was conducted for genes comprising MEs correlated with different MABC-PD groups. Network plot of genesets identified from (c) ME turquoise, (d) ME brown, (e) ME black, (f) ME royal blue, (g) ME blue, (h) ME red, (i) ME dark red, (j) ME yellow, (k) ME dark green.
(TIF)

S4 Fig Reduced NK and T cells abundance in patients on intensive phase treatment. Immune cell deconvolution using (a) quanTiseq and (b) MCP-counter. One-way ANOVA was used to test the difference in mean between patient groups. *p< 0.05, **p < 0.01. *n = 4* (intensive phase); *n = 3* (continuation phase); *n = 6* (watchful waiting); *n = 7* (post-treatment); *n* = 7 (healthy controls).
(TIF)

S5 Fig Box and whiskers plot comparing plasma concentration of proteins between patients on intensive phase treatment and healthy controls . Student’s *t*-test was used to test the difference in mean between the two groups.
(TIF)

S1 Data Raw data of protein measurements by Olink Target 48. Data provided include plasma concentration of proteins in pg/ml.
(XLSX)

S2 Data Subject attributes. Comprises subject number and their respective treatment/patient group used for RNA-sequencing analysis.
(CSV)

S3 Data Raw counts of gene expression data by RNA-sequencing. Data provided depict raw counts of gene expression data that was used as input for DESEq2 analysis.
(CSV)
