# Supplementary figures and images for: Transcriptional and cytokine signatures of Mycobacterium abscessus complex pulmonary disease during disease progression and treatment
Source: PLoS Negl Trop Dis. 2025 Mar 31;19(3):e0012943. doi: 10.1371/journal.pntd.0012943 (PMC11981118; doi:10.1371/journal.pntd.0012943)

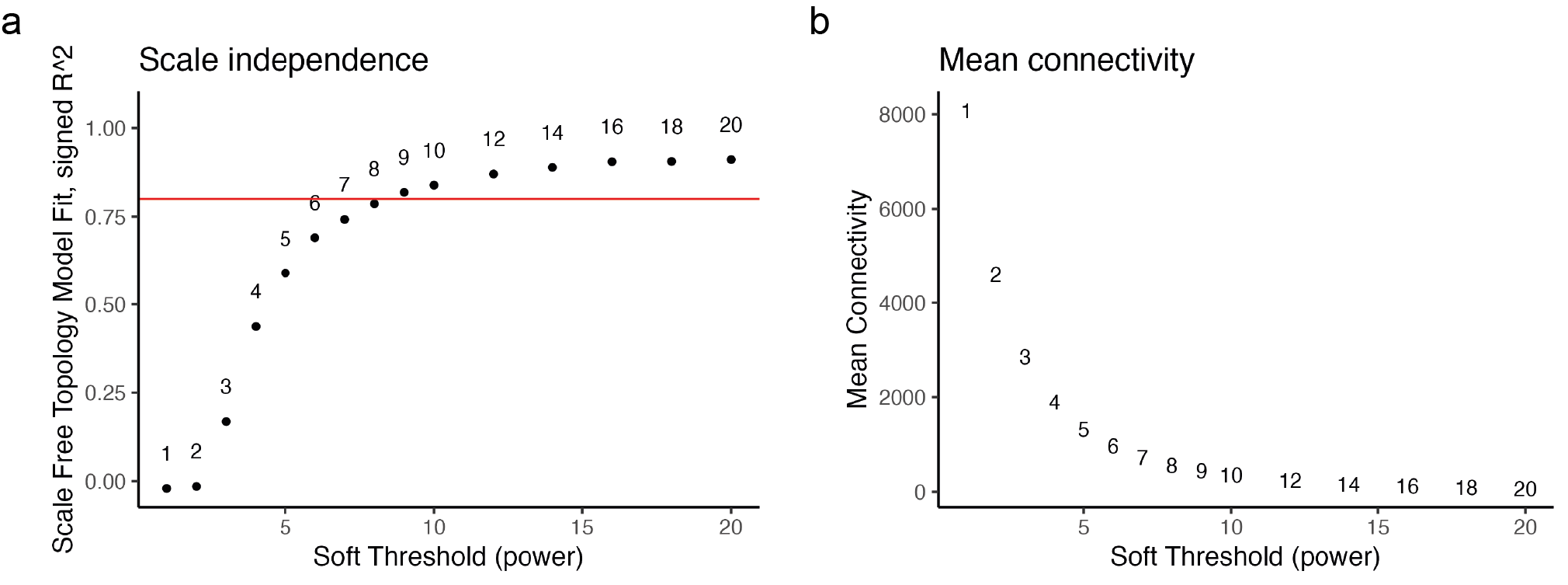

Supplement: S1 Fig — Diagnostic plots depicting (a) scale independence and (b) mean connectivity in determining soft threshold to be applied to WGCNA. (TIF) [file pntd.0012943.s003.tif]

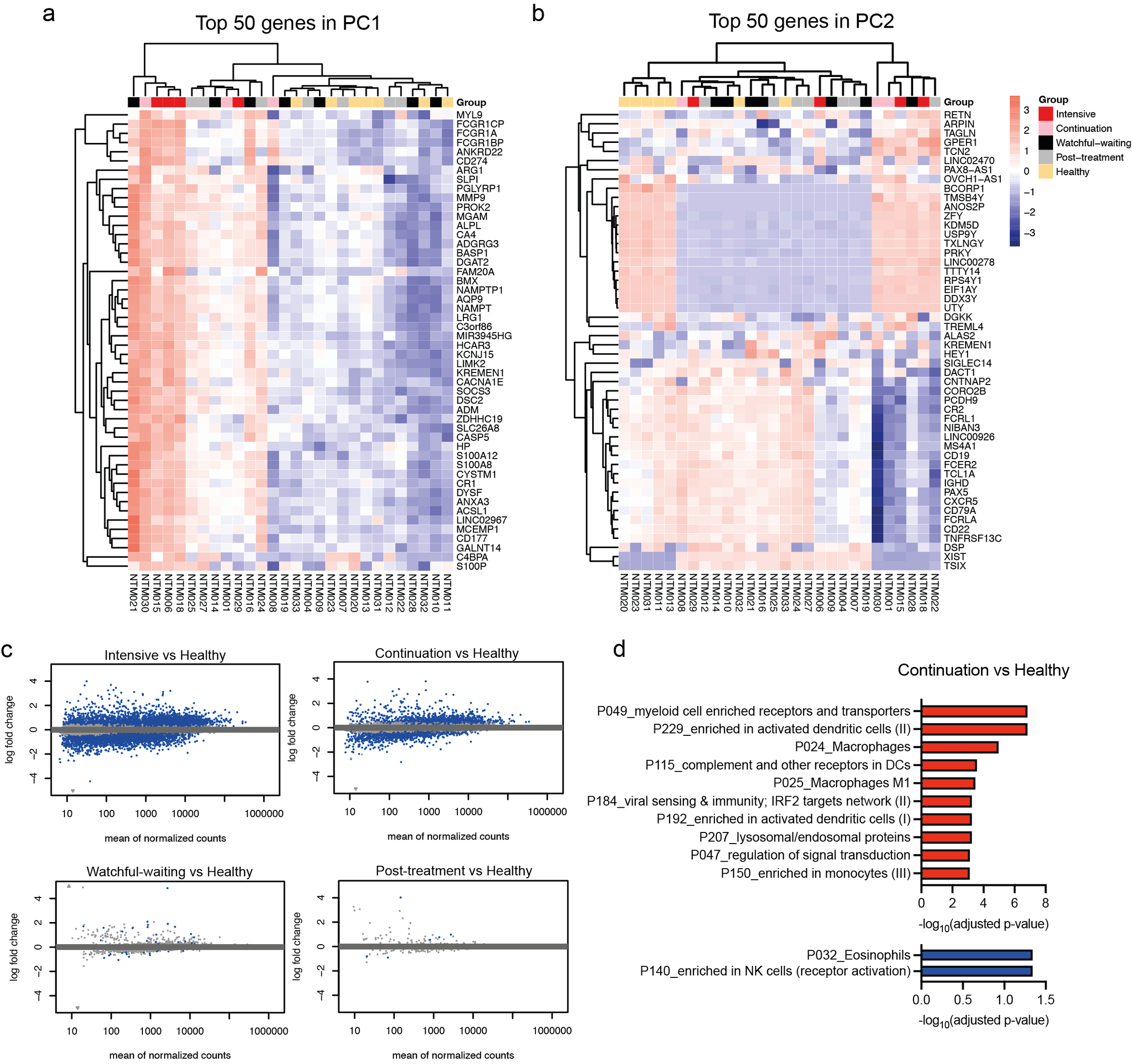

Supplement: S2 Fig — (a-b) Heatmap of the top 50 genes in (a) PC1 and (b) PC2. Each column represents a single patient in the study, rows represent genes. Different phases of MABC-PD treatment and disease progression are depicted in different colours. Z-scores of normalised counts are presented. (c-f) MA-plot depicting the mean of normalized counts across all samples for each gene and the log-fold-change between MABC-PD patients (c) on intensive phase treatment, (d) continuation phase, (e) on watchful waiting, and (f) post-treatment relative to healthy controls. (TIF) [file pntd.0012943.s004.tif]

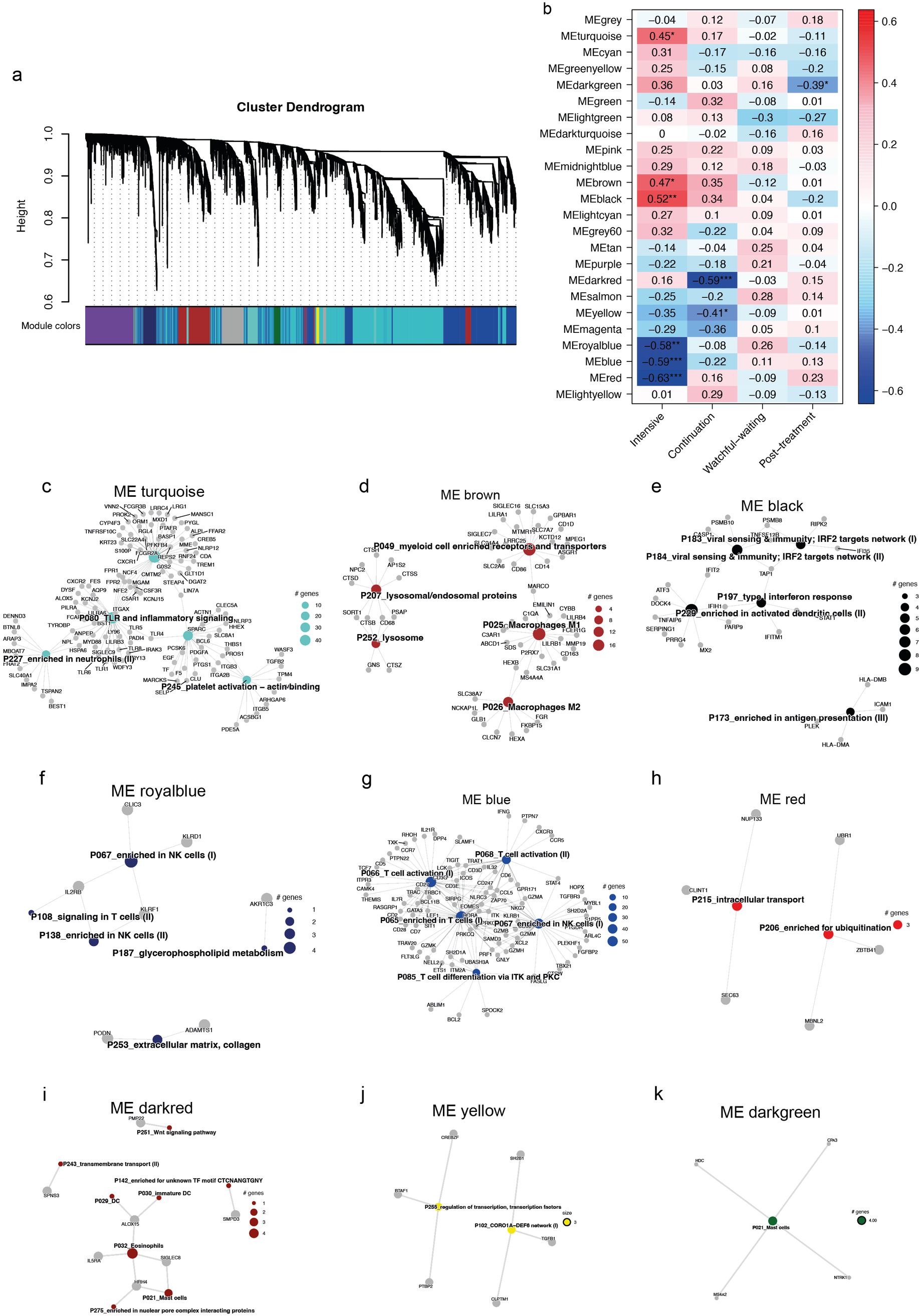

Supplement: S3 Fig — (a) Gene dendogram obtained by hierarchical clustering of dissimilarity based on Topological Overlap. (b) Heatmap of weighted gene co-expression network analysis (WGCNA) module-trait relationship, with trait being different treatment phases of MABC-PD. Rows represent module eigengenes that summarize gene modules defined in the hierarchical clustering analysis. Columns represent the different traits (i.e., treatment phases of MABC-PD). Values indicated in the box are the correlation coefficients with asterisks indicating the p-values. (c-k) Enrichr geneset pathway enrichment analysis using the BTM-plus was conducted for genes comprising MEs correlated with different MABC-PD groups. Network plot of genesets identified from (c) ME turquoise, (d) ME brown, (e) ME black, (f) ME royal blue, (g) ME blue, (h) ME red, (i) ME dark red, (j) ME yellow, (k) ME dark green. (TIF) [file pntd.0012943.s005.tif]

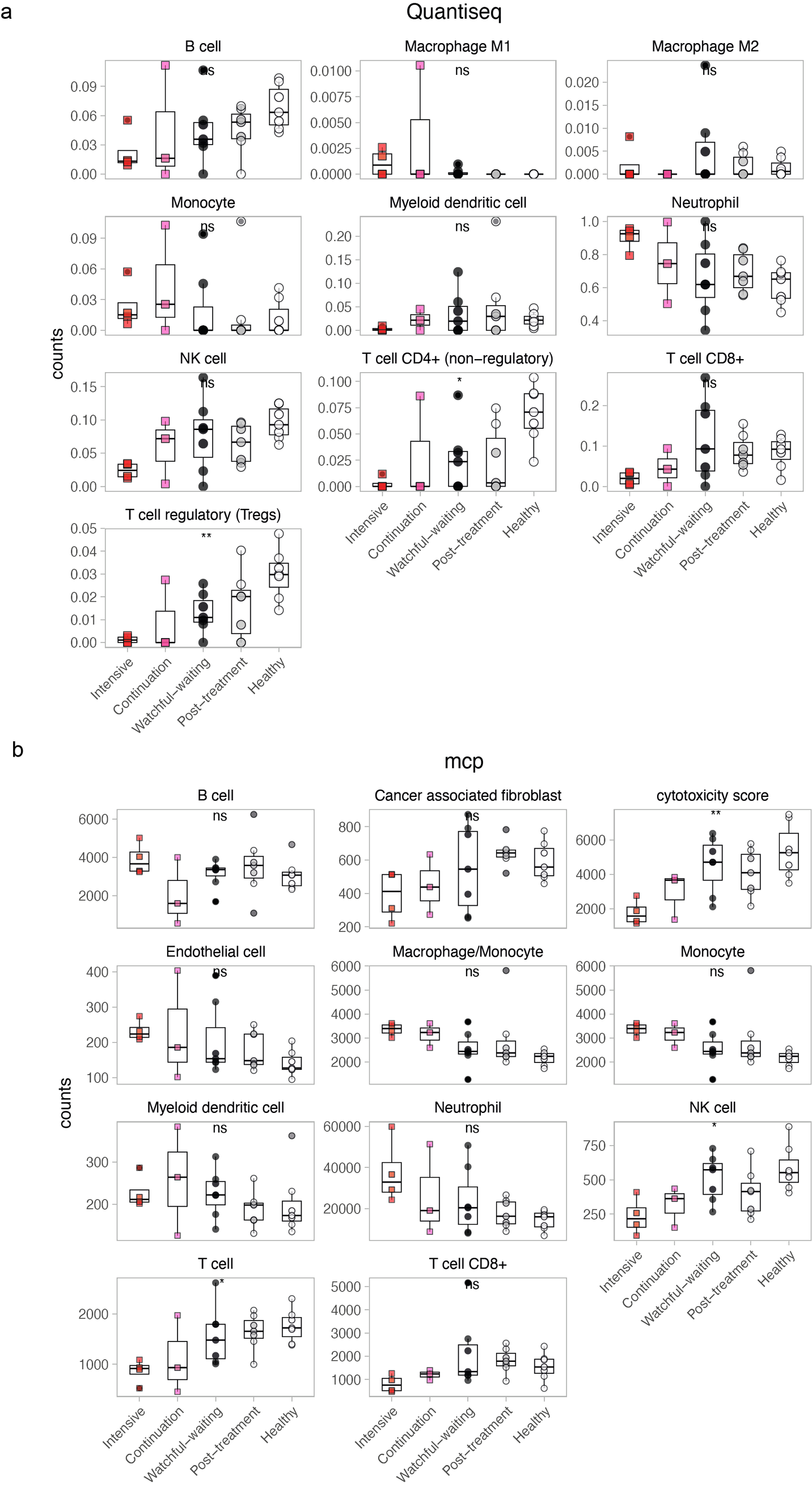

Supplement: S4 Fig — Immune cell deconvolution using (a) quanTiseq and (b) MCP-counter. One-way ANOVA was used to test the difference in mean between patient groups. *p< 0.05, **p < 0.01. n = 4 (intensive phase); n = 3 (continuation phase); n = 6 (watchful waiting); n = 7 (post-treatment); n = 7 (healthy controls). (TIF) [file pntd.0012943.s006.tif]

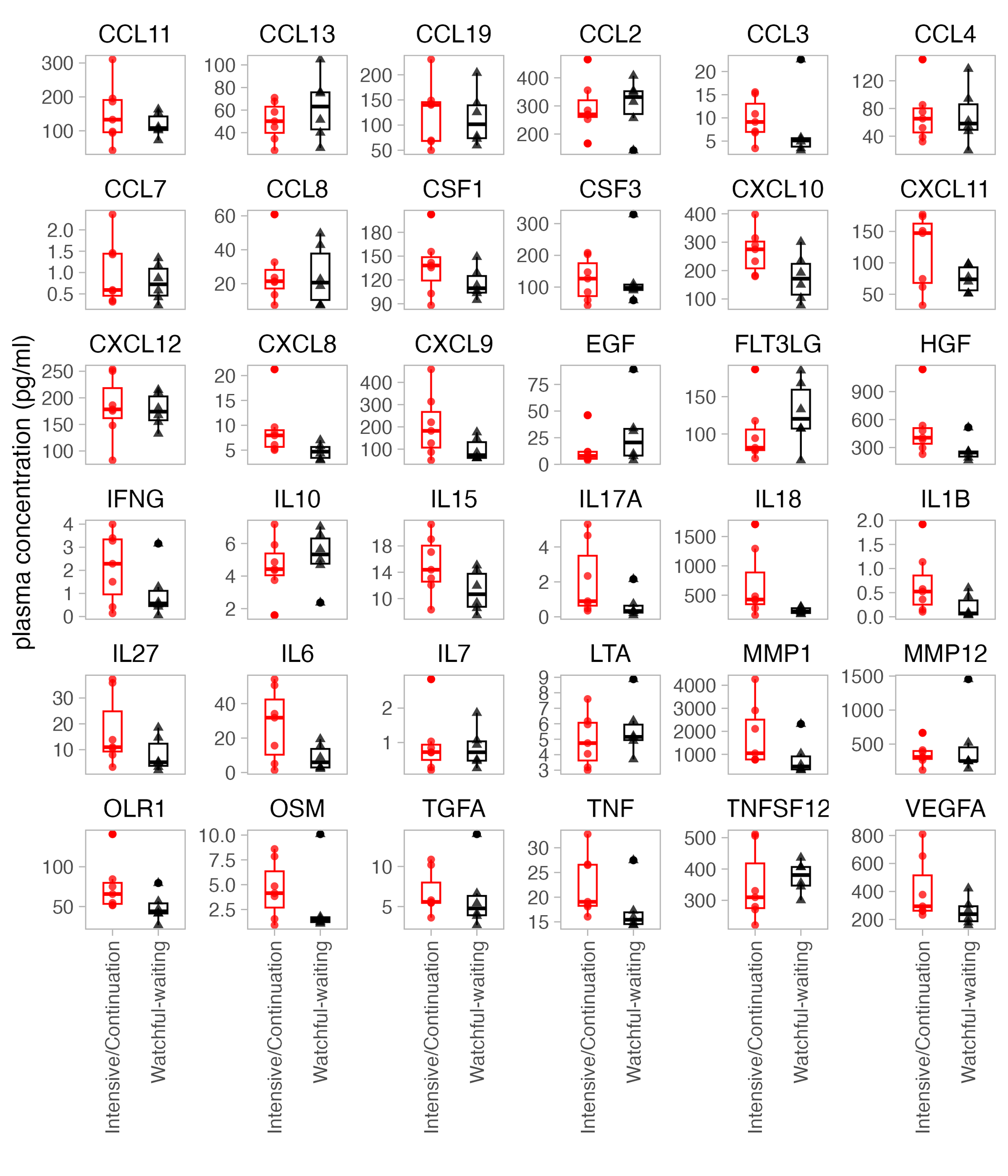

Supplement: S5 Fig — . Student’s t-test was used to test the difference in mean between the two groups. (TIF) [file pntd.0012943.s007.tif]
